# Career Intentions and Their Influencing Factors Among Medical Students and Graduates in Peshawar, Pakistan: A Cross-Sectional Study on Brain Drain

**DOI:** 10.7759/cureus.48445

**Published:** 2023-11-07

**Authors:** Zainab Tariq, Ameena Aimen, Unaiza Ijaz, Kashif Ur Rehman Khalil

**Affiliations:** 1 Community Medicine, Khyber Medical College, Peshawar, PAK

**Keywords:** medicine, intentions, physician migration, pakistan, brain drain

## Abstract

Background and objective: Physician brain drain is of mounting concern worldwide, especially in lower-middle-income countries like Pakistan, where the healthcare sector is overworked, and the exodus of talented health professionals further deteriorates the country's health statistics. Our study's objective was to investigate the career and migration intentions of medical undergraduates and graduates in Peshawar.

Design, settings, and participants: A cross-sectional self-structured questionnaire was distributed among 305 fourth- and final-year medical students and demonstrators at Khyber Medical College and graduates working as house officers, medical officers, and training medical officers at its conjugate hospital, Khyber Teaching Hospital, Peshawar. The questionnaire consisted of four sections designed to collect demographic details, determine participants' career and migration intentions, evaluate reasons for and against migration, and assess their reasons for establishing these intentions. The data were analyzed using IBM Corp. Released 2011. IBM SPSS Statistics for Windows, Version 20.0. Armonk, NY: IBM Corp.

Results: 67.5% of our respondents intended to migrate abroad, most of whom were men. The UK emerged as the top destination for those wishing to relocate. The most common reasons participants wanted to migrate were better quality of training and research, followed by a gain in professional skills over others, and lastly, better remuneration abroad. Most of those who wished to stay back chose family ties, a desire to serve the nation, and fixing flaws in the country's healthcare system as the most influential factors behind their decision to stay back.

Conclusion: The threat of brain drain is far-reaching and profound, putting Pakistan's people's health at risk. Policymakers must act to address the country's healthcare workers’ concerns.

## Introduction

One's acceptance into medical school in Pakistan is met with a pat on the back, followed by a celebratory greeting: "Are you migrating overseas after graduation?" Medical students and graduates in Pakistan are subjected to such inquiries incessantly. When they may not have given it a thought, the notion of moving abroad is coined to them, coupled with countless justifications, as if becoming a physician and transferring overseas go hand in hand.

This encouraged mass migration of proficient individuals has led to a phenomenon known as brain drain, a term coined by the Royal Society of London to describe the outflow of intellectuals to the United States and Canada in the early 1950s. This expatriation includes health practitioners, as they have always been subject to the lure of migration [1].

Today, accomplished physicians leaving their country to live or work for another in pursuit of improved quality of life are of mounting concern globally. In the US, the UK, Canada, and Australia, approximately one-quarter of practicing physicians are foreign-trained, and 40%-75% are from lower-income countries (LICs) [2]. This causes a colossal loss in financial resources and the drift of talented, aspiring individuals, devastating a nation's ailing citizens.

The loss of physicians to migration is costly for lower-middle-income countries (LMICs), with Saluja S’s study estimating that LMICs lose US $15.86 billion annually because of physician migration to higher-income countries (HICs) [3].

These figures translate into a loss of substantial national resources since public investment in healthcare providers in resource-poor countries tends to be greater than in more affluent ones [4]. On that account, developing nations are paying for the health care of developed nations, consequently steering considerable disparities in the global health workforce and furthering an economic divide between developing and developed countries. This repositioning of wealth is manifest in South Africa, where its overall monetary loss to high-income countries is US $1.41 billion while also being one of the highest HIV prevalence regions of the world [5].

With the increasing number of intelligentsia in LMICs bidding farewell to their countries, losing valuable human capital is perhaps one of the most staggering costs to pay. For instance, the approximate percentage of international medical graduates (IMGs) from LMICs working in the UK is an alarming 75.2%, followed by 60.2% in the US [2]. Consequently, many LMICs suffer from severe health staff shortages, as is reported, with HICs having a physician density of 300/100,000 and LICs having an unsettling 17/100,000 [6]. This health-worker shortage has especially hit India, Nigeria, Pakistan, and South Africa, resulting in a considerable socio-economic deficit, leaving these nations even more vulnerable with already fragile health systems [3].

Brain drain has severe implications for Pakistan. It stands on the fifth rank among the highest population in the world; however, by spending 38 US dollars on healthcare per capita, with only half of its 224 million population having access to healthcare, it is far behind in attaining the global UHC Service Coverage Index target of 80+ by 2030 [7,8]. This gap between the demand and the provision of health services may correspond to brain drain since Pakistan already holds an emigration factor of 11.7%, and the rate of migration has escalated over the years, with 14,352 IMGs of Pakistani origin working in the US in 2019 [2,9].

Our country is also facing colossal disease challenges. According to the Institute of Health Metrics and Evaluation (IHME), the annual rate of disability-adjusted life years (DALYs) lost was 42,059 DALYs/ 100,000 population in 2019, indicating that Pakistan has one of the highest burden of disease (BOD) in the region [7].

As per the disease groups' breakdown, neonatal problems account for the most considerable portion of BOD, with neonatal and infant mortality rates accounting for 42 deaths and 62 deaths per 1000 live births, respectively [7]. However, every prospect of resuscitating these tragic figures abates, with more and more health workers leaving.

Although there has been an increase in human resources from 2014 to 2021, the volatile political state affairs in Afghanistan meeting Pakistan on the western border have led to a massive influx of refugees and could subsequently expedite a dip in the ratio [7,8]. As per the latest statistics for 2021, 1.4 million Afghan refugees are registered in the country, with 58% residing in the province of Khyber Pakhtunkhwa (KPK), where our study was conducted [7]. These statistics translate to more than a million people requiring healthcare, and with emigration draining a valuable portion of our physicians and heavily burdening the system. Our study's objective was to determine the prevalence of external migration intentions of clinical medical students and junior doctors and their etiology by examining attitudes influencing these intentions to explicate the extent of physician migration from Peshawar.

## Materials and methods

Study design and setting

This cross-sectional study was conducted at Khyber Medical College and its primary teaching affiliate, Khyber Teaching Hospital, in 2022. The Ethical Review Board of Khyber Medical College approved the study. Informed consent was obtained from participants. Data was collected from the participants in two weeks, from May 30th to June 10th, through a self-administered questionnaire. 

Our inclusion criteria included consenting medical students from the fourth and final year medical students and medical graduates working as demonstrators, house officers, medical officers, and training medical officers. Participants were approached after lecture sessions and in hospital wards. We confirmed the total population size of our target population from Khyber Medical College's Medical Education Department.

Dual and foreign nationals were excluded from the study. Individuals having an exam following the month of data collection were not included either.

Sample size calculation

The sample size was calculated using OpenEpi, version 3, as follows:

n = [Np(1-p)]/ [(d2/Z21-α/2*(N-1)+p*(1-p)]

where

n = Sample size

N = Population size = 1470 confirmed from the Department of Medical Education at Khyber Medical College

p = Hypothesized % frequency of the outcome factor in the population, which is taken as 40% from a previous study [8].

d = margin of error= 5%

The sample size calculated from the above data was 295 participants. We were able to collect data from 305 participants. Our study tool consisted of a structured, self-administered questionnaire developed through an extensive literature search and input from the Department of Community Medicine at Khyber Medical College. The questionnaire was constructed in English and pilot-tested on the college’s faculty, who had prior experience in research, and the necessary changes were implied before finalizing it.

Data collection and analysis

Our branching-type survey had four sections. Section A asked about the participants' demographic profile (age, gender, year of education/occupation, relationship status), followed by their socio-economic standing (monthly salary and assets). Section B included variables pertinent to one's decision to emigrate, such as choice of specialty, international clinical experience, any relatives settled abroad, and their intention to migrate. It also asked about the participants' views on outmigration being a loss to Pakistan and if they would recommend others to emigrate post-graduation. Finally, those answering 'yes' to the intention to migrate were directed to Section C, which included their preferred choice of the destination country, their period of stay, and a total of 10 factors to choose from that could contribute to their intending to migrate. Those answering 'no' to the intention to migrate were asked to fill out Section D, which asked them to choose from several reasons to stay behind. This section's final part asked for recommendations for improving physician retention.

Data were entered and analyzed using IBM Corp. Released 2011. IBM SPSS Statistics for Windows, Version 20.0. Armonk, NY: IBM Corp. We used descriptive statistics, including frequencies, mean, and standard deviation, and the Pearson chi-square test to analyze different variables correlating with one’s intention to migrate. Chi-square tests were also applied to find a positive correlation between reasons for migration, retention, and recommendations in correspondence to gender. The p-value for significance for all was taken as <0.05.

## Results

There were 305 respondents from the study, out of which 152 were undergraduate students (male: 88; female: 64) and 153 were postgraduates (male: 90; female: 63). The participants' ages ranged from 20 to 36 years, with a mean age of 25.27 years (+/-2.977). The demographic information/profile of the participants are demonstrated in Table 1. Briefly, most of the respondents were single (74%), and 48% of the participants came from an urban background. 40.9% of the sample size had a monthly income ranging from 50,000 to 100,000 Pakistani rupees, and 52.4% claimed they had sufficient assets. An ample population (i.e., 62.9%) of the respondents' parents had a postgraduate degree, and 28.9% of people had a relative settled abroad. 73.4% of the respondents agreed that outmigration is a loss to the country; however, 64.2% still said they would recommend others to migrate overseas. It is noted that gender and international living experience are the only two variables positively associated with the participants' intention to migrate (p<0.05). This signifies that despite the participants' diverse backgrounds, one's intention to relocate is highly subjective and is impacted by other factors.

**Table 1:** Association between participants’ demographic profile and their migration intentions * 23 missing values, with three in parental education status, three in personal/family monthly income, and 17 in assets.

Demographic profile		Intention to migrate			
		Yes	No		p-value
Gender	Male	133	45		0.002
	Female	73	54		
Place of birth	Urban	96	51		
	Semi-urban	33	13		0.677
	Rural	77	35		
International living experience	Yes	47	12		0.027
	No	159	87		
Educational status	4th/5th year medical student	104	48		
	Medical graduate	102	51		0.744
Relationship status	Single	156	70		
	Engaged	15	10		0.589
	Married	35	19		
Parental educational status*	No formal schooling	15	6		
	Elementary	6	2		
	High school	32	12		0.821
	Undergraduate	26	11		
	Postgraduate	125	67		
Personal/family monthly income*	<50,000	7	2		
	50,000-100,000	82	43		0.658
	100,000-300,000	79	39		
	>300,000	37	13		
Assets*	Insufficient	23	6		
	Considerable	57	24		
	Sufficient	100	60		0.252
	Surplus	13	5		
International clinical experience	Yes	18	4		0.138
	No	188	95		
Family member settled abroad	Yes	66	22		0.076
	No	140	77		
View on outmigration being a loss to Pakistan	Yes	137	87		0.000
	No	69	12		
Recommendation for migration to others	Yes	172	24		0.000
	No	34	75		

The most preferred choice of specialty was internal medicine (31.1%), followed by cardiology (15.4%), general surgery (8.2%), gynecology (7.5%), and pediatrics (7.5%), constituting a cumulative percentage of 69.7%. Interestingly, four out of five of these specialties were also the respondents' preferred choices in another study done in Pakistan [10].

Participants intending to migrate

**Figure 1:**
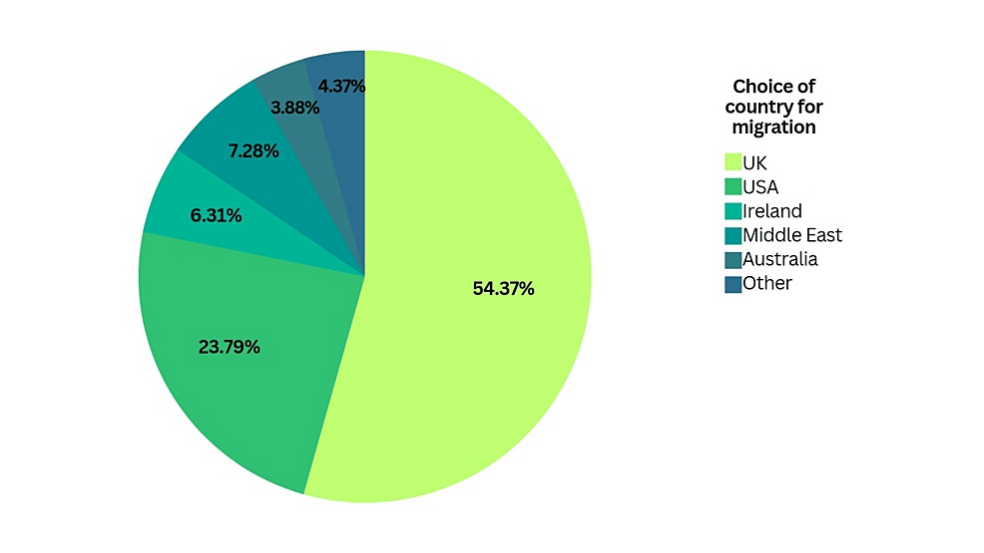
Participants' choice of country for migration

An overwhelming majority, i.e., 209 of the 305 participants, intended to migrate abroad. The UK emerged as the top destination for our participants, with a response rate of 53.5%, followed by the US with 23.4%. Of the remaining, 7.1% wanted to migrate to the Middle East, 6.2% to Ireland, 3.2% to Australia, and 4.3% chose 'other.' This is portrayed in Figure 1.

Furthermore, 81.8% wished to emigrate for specialty training, while 18.1% wished for a locum job. 68.8% of the respondents wanted to stay in the host country temporarily upon migration, while 19.6% wanted to move permanently. Finally, 10% of the participants were unsure how long they would remain in the host country. These statistics are displayed in Table 2.

**Table 2:** Variation among participants intending to migrate

		Gender		
		Male	Female	p-value
Migration purpose	Locum job	28	7	0.036
	Specialty training	105	66	
Period of stay	Temporarily	91	53	
	Permanently	28	13	0.816
	Not sure	14	7	

**Figure 2:**
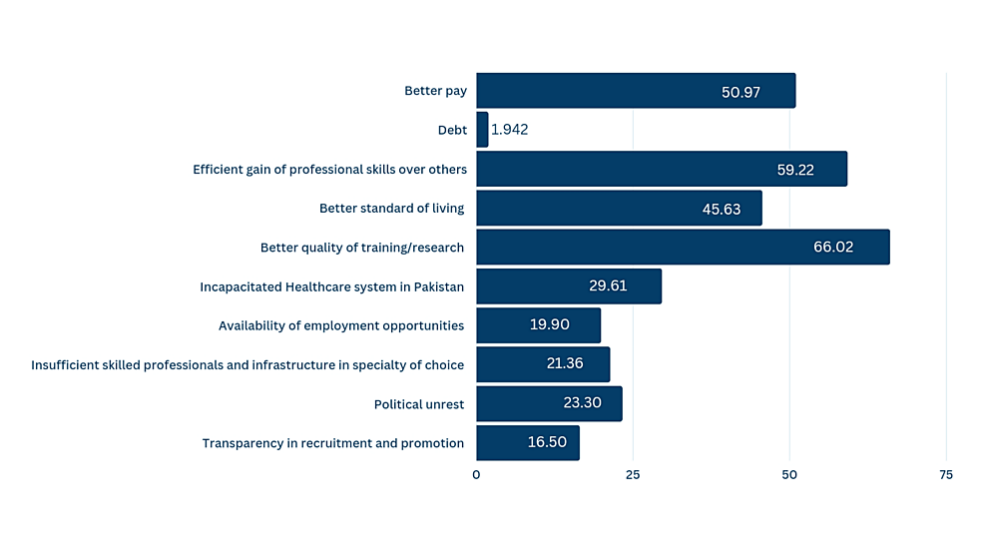
Participants' reasons for migration

Figure 2 shows the respondents' motivations to migrate abroad. Better quality of training and research (65%), an efficient gain of professional skills over others (58.3%), and a better salary (50.2%) were the leading influencing factors for emigration. In comparison, the least influencing factor was debt (1.9%).

Participants intending to stay back

Ninety-nine of the 305 participants intended to stay back in Pakistan. The major factors behind the participants' intention to stay back included family ties (72.7%), desire to serve the nation (60.6%), and fixing flaws in the country's healthcare system (41.4%).

In the last section of the questionnaire, those intending to stay back were invited to recommend changes to reduce emigration. Most of the 99 respondents reported that improved administrative policies (73.7%) and quality of training (70.7%), along with transparency in recruitment and promotion (55.5%), need to be introduced to reduce outmigration. Table 3 shows other factors behind the participants' decision to stay back and their suggestions for retention.

**Table 3:** Response of participants intending to stay back in Pakistan

Reasons for staying back in Pakistan			
	Gender		
	Male	Female	p-value
Family ties	36	36	0.138
Desire to serve Pakistan	22	38	0.029
Fix flaws in current healthcare system	19	22	0.882
Religious factors	12	15	0.902
Adequate/alternative financial support	9	6	0.219
Lack of finances to fund settlement abroad	11	6	0.080
Complete confidence in the current healthcare system	3	5	0.637
Job satisfaction	4	16	0.010
Visa problems	1	0	0.271
Recommended changes to encourage staying back in Pakistan			
Improved administrative policies	33	40	0.934
Improved quality of training	30	40	0.420
Introduction of unappreciated specialties’ departments	19	24	0.824
Transparency in recruitment and promotion	23	32	0.417
Provision of career counselling	13	30	0.008
Increased incentives/salaries	25	28	0.713
More professional development opportunities	19	26	0.555
Political stability	15	13	0.308
Job security	18	34	0.023
None	2	0	0.118

## Discussion

This study aimed to determine the emigration potential of undergraduates and junior doctors in Peshawar, Pakistan, with a mean age of 25.3 years (std 2.98). From our findings, 67.5% of the participants intended to migrate abroad. These results are analogous to the emigration intentions of several other Pakistani colleges, like Baqai Medical College (65%), Dow Medical College (60.4%), and King Edward Medical University (60.4%) [10-12]. Aga Khan University, however, had a much higher emigration prevalence rate (95%) [11]. This difference may be attributed to Aga Khan University being a private medical college with much higher expenses in Pakistan. Our findings were consistent with data from other countries like Ireland and Nigeria [13,14]. A study of 1214 students in Poland also resulted in 62.1% of the respondents planning to seek employment abroad after graduation [15].

In our analysis, the UK emerged as the most popular destination for immigration, closely followed by the US. A study from India displayed a similar pattern, with most of the respondents ranking the UK as their top destination for emigration, albeit with a smaller difference in choice than our participants [16]. However, these findings differed from those found in research at other Pakistani medical institutions, where students preferred the US over the UK [10,12]. Factors such as less preparation time and finances required for the PLAB and comparatively smoother matching into a hypercompetitive specialty in the UK might have been of influence [17].

Significantly, 83% of our migrating population intended to move overseas for specialty training over locum jobs. We also observed a similar trend in a study conducted in Lahore, Pakistan, where 54.9% of their migrating participants planned to migrate for professional reasons, signifying that these respondents were more inclined to migrate to excel professionally than for monetary gain [12]. Out of the small population (7%) wanting to migrate to the Middle East, 80% chose locum jobs over specialty training, with four times more men than women. Literature generally associates locum migration with financial prosperity, signifying that migration to the Middle East is associated with good salary packages but is not preferred to gain a professional advantage.

The demographic profile of our respondents reported gender and international living experience as significant in correlation with their intent to migrate. We observed that people without international living experience (77.18%) and a higher percentage of males (64.6%) intend to migrate abroad. The bifurcation of gender with more women choosing to stay back may be attributed heavily to our patrilocal society, traditional cultural values, and ossified gender roles in the country. These gendered ramifications are observed especially in KPK, where a woman's decision to migrate is heavily influenced by her parents, spouse, in-laws, and socially rooted gender roles as homemakers and mothers. Another factor that may be contributing to more men migrating is years of conflict and political instability in the country, as is seen in Ethiopia, mirroring our results, while Sri Lanka contradicts them, attributing this difference to Sri Lanka emerging from a war to a peaceful country in 2009 while the former is still a victim of political unrest [18]. Pakistan’s political misfortune may be elicited by the fact that a prime minister has never completed a full term in office. With yet another prime minister ousted in 2022, another setback due to recent political and economic instability in Pakistan is the interrupted continuity of the Sehat Sahulat Program (SSP), Pakistan's first health insurance initiative. While it is still in service in some parts of the country, SSP has been discontinued in others [8]. Zahid Hussain's 'Can political stability hurt economic growth?' reports a deep interconnection between hostile political environments and the hampered pace of economic development [19]. This correlation is manifest in the country through the volatile political affairs owing to the rampant devaluation of the Pakistani rupee this year. The precarious economic state of affairs in the country puts more pressure on men than women since Pakistan’s traditions hold men as breadwinners in a household. A Tunisian study supports this predicament, reporting more men migrating for economic reasons compared to women [20]. Interestingly, only 23.3% of our study population chose political unrest as a motivating factor, as compared to other studies with dismal political conditions as a major driving force [18,21,22].

The top four factors reported in our study as being influential to our participants' decision to migrate are as follows: better quality of training and research, gaining professional skills, a better salary abroad, and the incapacitated healthcare system in Pakistan. Aly, Z., and Taj, F.'s study [23] and reports from several Sub-Saharan countries, Nepal, the Philippines, and many more, delineate similar reasons [21]. These factors owing to migration are described in ascending order, from the most influential discussed first to the least influential discussed last.

Our intended population's primary reason behind emigration was a better quality of training and research, with a response rate of 65%. This determinant was also cited as the primary reason by the Aga Khan University and Baqai University sample populations in Pakistan [11]. Moreover, HICs have ample opportunities for IMGs to offer well-structured training and research prospects even in lesser-explored specialties like neonatology and child psychiatry, the latter having only one unit in a tertiary care hospital in the province of KPK. With a saturated job market, better quality of training puts one a step ahead. Thus, migration plans to gain a professional advantage (58.3%) closely following better training quality were not surprising to see. The desire to excel professionally was also documented in several other studies [21].

Better remuneration in the destination country also coaxes physicians to emigrate, with 50.3% of our participants choosing this as one of the factors. Pakistan's public health expenditure in FY 2020-21 reserved for health was a meager 1.2% [24] as compared to Egypt’s 5.5% [25] and Libya’s 3.3% [26], which is seemingly low compared to what the World Health Organization has recommended. Consequently, HICs offer higher pay, as seen by a physician in the US earning nearly $339,000 as of 2022 [27]; in Pakistan, a physician of identical caliber can barely make half that amount. The paucity of salaries matching the study population's talent proves to be a significant driver for emigration and is also cited in several other studies [10,11,14,15,18,22,26]. Twenty nine percent of our study population chose an incapacitated healthcare system, accounting for their migration decision. With the current predicament of rising inflation and overpopulation, Pakistan's public healthcare systems are left enervated. The pronounced underinvestment in the health sector, poor utilization of available resources, insubstantial directorial aptitude, poor staffing, and abandoned initiatives for the convalescence of the healthcare system have led to poor self-esteem and increased stress in doctors [28]. Since better healthcare resources attract physicians, countries rewarding physicians in accordance with their skills and abilities lure Pakistani doctors to expatriate.

We found that 69.9% of the respondents wished to migrate temporarily and planned on returning to the country. Many studies conducted in developing nations have cited similar results [15,18]. Literature indicates having strong personal and family roots in a native country is a good predictor of retention and return [18,21,22]. Abraham Maslow also describes the importance of forming a family unit and a sense of belonging, referring to the human need for social connectedness. Therefore, factors such as family relations, living with old-age parents, or raising a family in their home country could be a reason for people to return. However, this desire to be with family can lead to one deciding not to migrate, as seen in our study, as 72.7% of our respondents staying back in Pakistan chose family ties as their primary reason for staying back. Another study also reports similar results, where respondents expressed deep gratitude for staying home, raising children in their native religion and culture, and having the opportunity to tend to their parents while acknowledging their country's failings [29]. The second most common reason reported by our respondents for staying back in Pakistan was their desire to serve the country and its people (60.6%). We observed the same theme recurring in studies from Aga Khan University and Sri Lanka [11,18]. Almost every nation invests tax money in producing quality doctors. Therefore, our participants might feel a sense of moral obligation to help people in their community, leading to it being one of the influencing factors behind doctors choosing to stay back. 41.4% of those intending to stay back reported that they wanted to fix flaws in the current healthcare system, portraying that Pakistani doctors are unanimous that our healthcare system calls for marked rectifications. Hence, the acknowledgment of the country's shortcomings concerning its healthcare system coupled with a will to rebuild it was a fairly common sentiment those choosing to stay back expressed.

Strengths and limitations

We researched a diverse population at a leading public-sector medical school and its attached teaching hospital in Peshawar. Unlike most research in low-resource environments that has focused on graduates and practicing doctors, our study involved both undergraduates and postgraduates. Therefore, our findings can help curate policies for medical students and young graduates. Second, we asked for recommendations from the students and junior doctors staying back. The perspectives and experiences of professionals who continue to practice in their home country know the system's failings well. Their experiences and insights could provide invaluable information on where to start rebuilding the country's health policy. Lastly, to the authors' knowledge, no such studies have investigated brain and skills drain in medicine in Peshawar.

There is a need for studies conducted on a larger scale covering various regions of Pakistan to offer more generalizable insights into the emigration patterns among medical professionals in the country. Additionally, a few questions were left unanswered by some participants, predisposing our study to non-response bias. Another limitation of our study was that convenient sampling was employed. However, we received almost equal responses from undergraduates and graduates (49.8% and 50.2%, respectively), and since it was an observational study, it did not adversely influence our results. Future studies could benefit from employing stratified random sampling or other methods that might yield a more representative sample. 

Recommendations

Promoting and rewarding credible professionals with a strong work ethic and establishing methodical old and new graduate programs in which trainees are well paid are some ways that can foster fulfilling careers in Pakistan. Gender and international living experience are two variables positively associated with the intent to migrate. This could have implications for gender-specific policies to address the brain drain issue. Furthermore, as demonstrated in our study, most migrant doctors want to return to their home country. Across the globe, there are numerous esteemed and renowned Pakistani researchers and physicians whose expertise and support can be invaluable if offered a structured return program, offering incentives such as senior positions, research opportunities, or positions in ministries or think tanks. Additionally, family ties and a desire to serve the nation are powerful deterrents to migration. It is crucial to understand how these influences can be harnessed to improve physician retention. 

In light of the factors causing physician exodus, health policymakers and academic institutions must make swift and brave decisions by re-establishing their priorities to harmonize with their country's needs. Instead of coercing doctors by lowering their ambitions and standards to serve in an environment below their credentials, our cardinal motive should be to make Pakistan a welcoming place where talented individuals aspire to stay and work.

## Conclusions

In this study, we observed the factors leading to a desire for migration among undergraduates and junior doctors in Peshawar, Pakistan. We compared various demographic and socio-economic factors with the participants' intent to migrate to find the reasons behind these intentions. We discovered that 67.5% of our respondents intended to emigrate owing to the dearth of opportunities hindering physicians from progressing professionally, healthier remuneration, and quality training and research opportunities abroad. The expatriation of physicians puts the future of Pakistan's healthcare sector at risk. Unless there is persevering determination and will, reflected in daring actions to make Pakistan more apt at owning and retaining its diverse talent and intellect, which could substantially ameliorate the country's fate, this pruning of physicians will continue to grow.
